# *Hexatoma (Eriocera)* Macquart (Diptera, Limoniidae) from Xizang, China

**DOI:** 10.3897/zookeys.1071.66750

**Published:** 2021-11-18

**Authors:** Bing Zhang, Qicheng Yang, Yan Li, Ding Yang

**Affiliations:** 1 Department of Entomology, College of Plant Protection, China Agricultural University, Beijing 100193, China China Agricultural University Beijing China; 2 Hubei Insect Resources Utilization and Sustainable Pest Management Key Laboratory, College of Plant Science & Technology of Huazhong Agriculture University, Wuhan, 430070, China Huazhong Agriculture University Wuhan China; 3 College of Plant Protection, Shenyang Agricultural University, Shenyang, 110866, China Shenyang Agricultural University Shenyang China

**Keywords:** Biodiversity, craneflies, Limnophilinae, systematics, taxonomy, Tibet

## Abstract

One new species of the subgenus Eriocera Macquart,1838, Hexatoma (Eriocera) xizangensis**sp. nov.** is described and illustrated from Xizang, China. The following four species are re-described and reported from Xizang for the first time: H. (E.) latigrisea Alexander, 1971, H. (E.) nepalensis (Westwood, 1836), H. (E.) paragnava Alexander, 1973 and H. (E.) perhirsuta Alexander, 1973. A key to the species of *Eriocera* from Xizang is presented.

## Introduction

Eriocera Macquart, 1838 is a subgenus of the genus Hexatoma Latreille, 1809 in the family Limoniidae. It is distributed worldwide with 563 known species and subspecies, of which 69 taxa are from the Palaearctic Realm, 34 taxa from the Nearctic Realm, 143 taxa from the Neotropical Realm, 30 taxa from the Afrotropical Realm, 290 taxa from the Oriental region, and five taxa from the Australasian/Oceania realms (Oosterbroek 2021). The subgenus is thus large and morphologically diverse, and was confirmed to be non-monophyletic by Ribeiro (2008). It is characterized by the following characters: body medium to large sized; antenna with four to ten flagellomeres; wings often unpatterned through variously darkened, or with a conspicuous hyaline and yellow cross banded pattern, or abundantly spotted and dotted with brown; cell *dm* present; two or three branches of M reaching margin; clasper of gonostylus narrowed apically into a long curved spine; lobe of gonostylus short and stout with setae; gonocoxite moderately stubby or elongate cylindrical; interbase usually cylindrical, or triangular with a sharp spine at base, or two-layered membranous structure with spine at apex; aedeagus usually short and relatively inconspicuous, or directed ventrally (Alexander and Lloyd 1914; Edwards 1921; Alexander 1948; Alexander and Byers 1981; Savchenko 1986; Podenas et al. 2006; Ribeiro 2008; Podeniene and Gelhaus 2015).

So far, only the subgenus Eriocera were known to occur in Xizang (Men and Yu 2015; Oosterbroek 2021): H. (E.) lanigera Alexander, 1933, H. (E.) mediofila Alexander, 1933, H. (E.) nudivena Alexander, 1933 and H. (E.) tibetana Alexander, 1933. To enrich the knowledge of the species composition of craneflies in Xizang, we conducted a scientific survey of craneflies in Xizang from 1978 to 2019. Presently, five species including one new species of the subgenus Eriocera are added to the fauna of Xizang. The following three species are reported from China for the first time: H. (E.) latigrisea Alexander, 1971, H. (E.) paragnava Alexander, 1973 and H. (E.) perhirsuta Alexander, 1973. Hexatoma (E.) nepalensis (Westwood, 1836) is for the first time reported from Xizang. Hexatoma (Eriocera) xizangensis sp. nov. is described and illustrated from Xizang. A key to the species of *Eriocera* from Xizang is presented.

## Materials and methods

The specimens were studied and illustrated with a ZEISS Stemi 2000-c stereo microscope. Details of coloration were checked in specimens immersed in 75% ethyl alcohol. Male genitalia were prepared by macerating the apical portion of the abdomen in cold 10% NaOH for 12–15 hours. After examination, it was transferred to fresh glycerine (C_3_H_8_O_3_) and stored in a microvial pinned below the specimen. The specimens studied, which were collected in Xizang are deposited in the Entomological Museum of China Agricultural University (CAU), Beijing, China.

Holotype material of Hexatoma (Eriocera) latigrisea Alexander, 1971 used in this paper was borrowed from the National Museum of Natural History, Smithsonian Institution, Washington, DC, USA (USNM) and holotype material of Hexatoma (Eriocera) nepalensis (Westwood, 1836) was borrowed from the Natural History Museum, London, UK (BMNH). The terminology applied to the wing veins follows the interpretation of de Jong (2017). Terminology of the male terminalia follows Ribeiro (2006, 2008). The following abbreviations in figures are used: 9s = ninth sternite, 9t = ninth tergite, goncx = gonocoxite, cgonst = clasper of gonostylus, lgonst = lobe of gonostylus, aed = aedeagus, ce = cercus, hy = hypogynial valve.

## Taxonomy

### Key to species of subgenus Eriocera from Xizang, China (adult)

**Table:** 

1	Wing with cell *M_1_* (Figs 34, 40)	**2**
–	Wing without cell *M_1_* (Figs 1, 3, 9, 10, 12, 16, 22, 24, 28, 46, 48)	**4**
2	Antenna of male approximately as long as body (Alexander, 1933: 150)	**H. (E.) tibetana Alexander, 1933**
– Antenna of male approximately three times as long as body (Figs 34; Alexander, 1973: 8; Alexander, 1933: 149)	**3**
3	Femora yellow (Figs 34; Alexander, 1973: 9); *R_2+3_* two times longer than *R_2+3+4_* (Figs 34, 40; Alexander, 1973: 4, fig. 6)	**H. (E.) perhirsuta Alexander, 1973**
–	Basal 1/3 of femora yellow, outer 2/3 black (Alexander, 1933: 149); *R_2+3_* as long as *R_2+3+4_* (Alexander, 1933: plate I; Fig. 11)	***H. (E.) lanigera Alexander* , 1933**
4	Wing without markings (Figs 22, 24, 28)	**5**
–	Wing bicolorous, with markings (Figs 1, 3, 9, 10, 12, 16, 46, 48)	**7**
5 Wing without stigma (Alexander, 1933: 158); *R_2_* longer than *R_2+3_* (Alexander, 1933: plate I. Fig. 19)	**H. (E.) nudivena Alexander, 1933**
– Wing with stigma; *R_2_* shorter than *R_2+3_* (Figs 22, 24, 28)	**6**
6	Antenna of male approximately as long as body (Alexander, 1933: 151); *R_2+3_* longer than *R_2+3+4_* (Alexander, 1933: plate I; Fig. 13)	**H. (E.) mediofila Alexander, 1933**
–	Antenna of male approximately three times longer than body (Figs 22; Alexander, 1973: 8); *R_2+3_* shorter than *R_2+3+4_* (Figs 22, 24, 28; Alexander, 1973: 4, fig. 5)	***H. (E.) paragnava Alexander* , 1973**
7	Male terminalia yellow (Fig. 46); wing with many hyaline markings; *R_2_* contacts vein *R_2+3+4_* (Figs 46, 48)	**H. (E.) xizangensis sp. nov.**
–	Male terminalia black or dark brown (Figs 1, 9, 10); wing with a whitened or hyaline marking before discal area; *R_2_* contacts vein *R_2+3_* (Figs 1, 3, 9, 12, 16)	**8**
8	Antenna blackish brown; femora blackish brown; abdomen of male with four pale white gray markings (Figs 9, 10) ...... **H. (E.) nepalensis (Westwood, 1836)**
–	Scape and pedicel blackish brown, flagellum reddish yellow except outer two segments dark brown; basal 5/6 of femora yellow, outer 1/6 dull black; abdomen glossy black or dull black (Fig. 1; Alexander, 1971: 116)	**H. (E.) latigrisea Alexander, 1971**


#### Hexatoma (Eriocera) latigrisea

Taxon classificationAnimaliaDipteraLimoniidae

Alexander, 1971

A0260828-ED4A-502C-9790-A6B7A4713D4F

Hexatoma (Eriocera) latigrisea Alexander, 1971: 116. Type locality: India, Assam

##### Specimens examined.

2 males (CAU), China: Xizang, Motuo, 80K, 2014.VII.31, Yan Li (light trap). ***Holotype***: male, Kujjalong, Kameng, North East Frontier Agency, Assam, 4500 feet, June 28–30, 1961 (Schmid).

##### Diagnosis

. Antenna has 8 segments. Wing is brown, but narrowly yellow at base; anal cells are much paler; cells *C* and *Sc* are dark yellow; a narrow white discal area is before cord, including cell *R_1_* to cell *M*; *m-cu* is nearly at middle of cell *dm*. Posterior margin of male ninth tergite has a V-shaped notch, both sides of notch have a process; interbase is triangular, swollen at base.

##### Redescription

. Male (*N* = 2): Body length 18.0–19.8 mm, wing length 14.0–16.7 mm, antenna length 3.6–4.1 mm.

**Figures 1–3.**
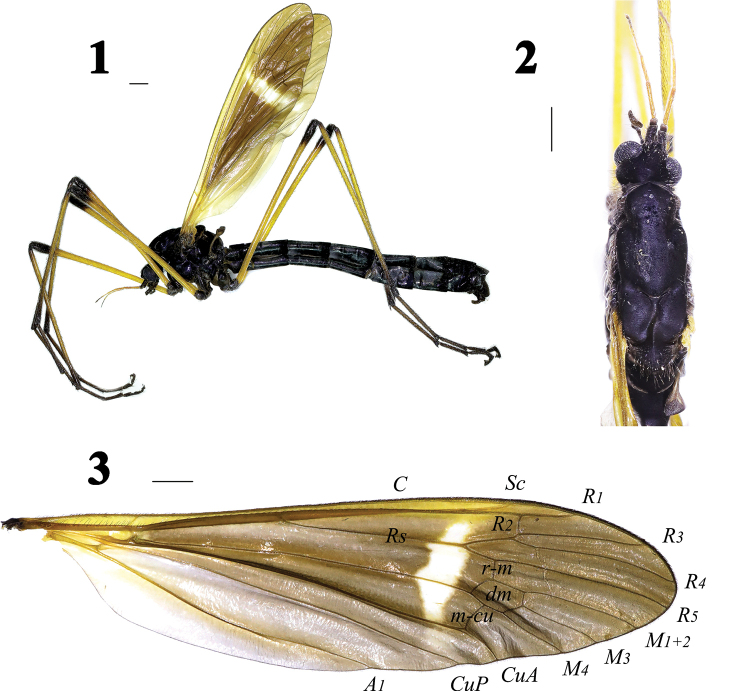
Hexatoma (Eriocera) latigrisea Alexander, male **1** habitus, lateral view **2** male head and thorax, dorsal view **3** right wing. Scale bars: 1.0 mm (1–3).

Thorax (Figs 1, 2) dull black with blackish brown setae. Legs with blackish brown setae; coxae and trochanters blackish brown; basal 5/6 of femora yellow, outer 1/6 dull black; tibiae obscure yellow, black apically; tarsi dark brown. Wing (Figs 1, 3) brown, narrowly yellowed at base; anal cells much paler; cells *C* and *Sc* dark yellow; a narrow white discal area before cord, including cell *R_1_* to cell *M*; veins brown, more yellow in brightened areas. Venation: *R_2_* moderately oblique, *R_2+3_* relatively short, shorter than *R_2_*; cell *M_1_* lacking; *m-cu* nearly at middle of cell *dm*. Halter (Figs 1, 2) length approximately 2.2 mm, halter stem pale brown with brown setae; knob brown with blackish brown setae.

Abdomen (Fig. 1) with short black setae. Segments 1–6 extensively glossy black, segments 7–8 dull black.

**Figures 4–8.**
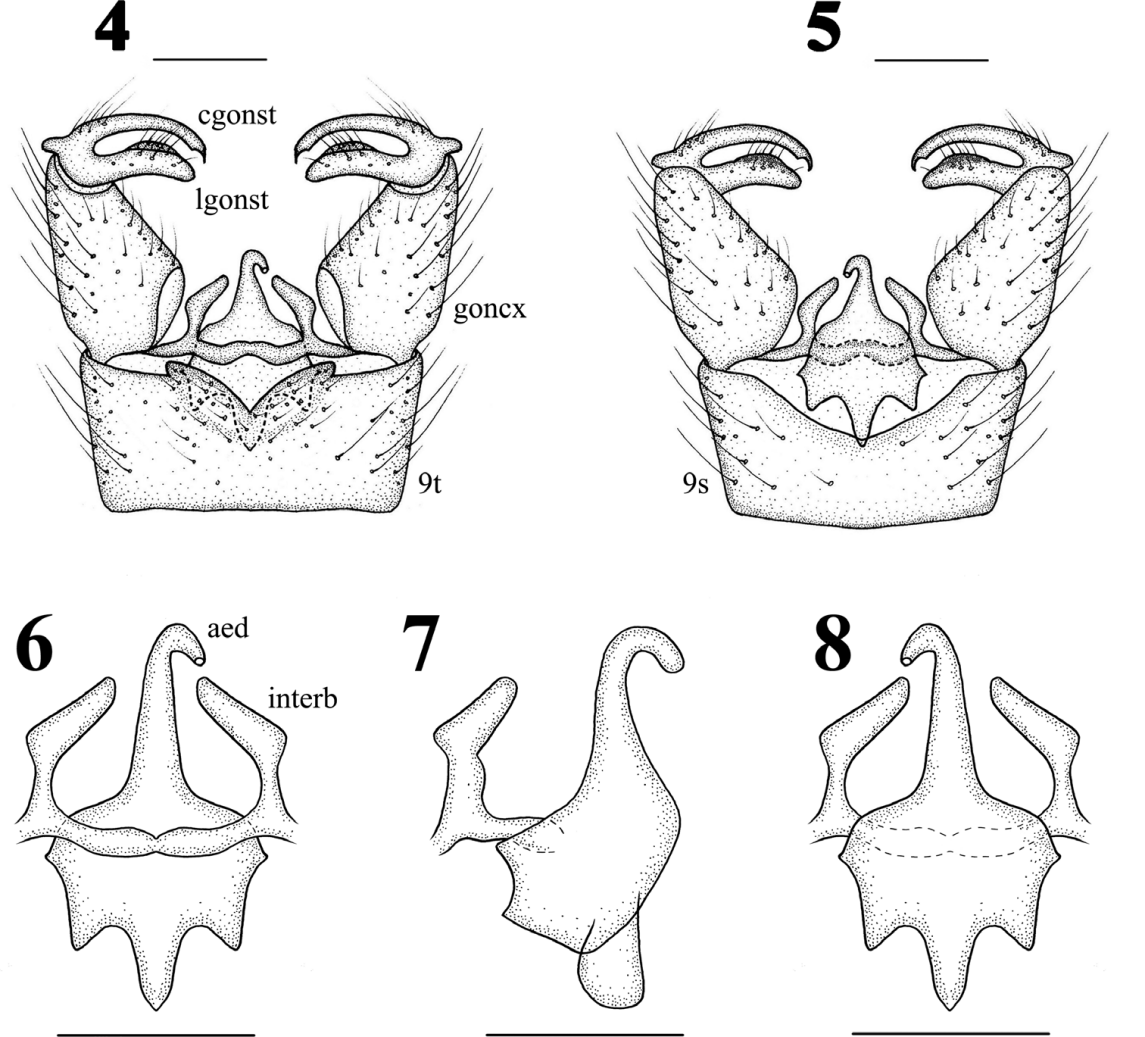
Hexatoma (Eriocera) latigrisea Alexander, male **4** terminalia, dorsal view **5** terminalia, ventral view **6** aedeagal complex, dorsal view **7** aedeagal complex, lateral view **8** aedeagal complex, ventral view. Scale bars: 0.5 mm (4–8).

Male terminalia (Figs 1, 4–8) with 180° rotation, dull black with black setae. Posterior margin of ninth tergite with a V-shaped notch, both sides of notch with a lateral projection; posterior margin of ninth sternite with a deep V-shaped shallow; gonocoxite moderately stubby; clasper of gonostylus with long setae at base, slender, terminal spine decurved; lobe of gonostylus short and stout, terminal margin swollen with long setae; interbase triangular; aedeagus longer, apically directed ventrally.

##### Female

. Unknown.

##### Distribution

. India (Assam), China (Xizang).

##### Remarks

. This species was known previously only from India. With the present contribution it is recorded from China for the first time.

#### Hexatoma (Eriocera) nepalensis

Taxon classificationAnimaliaDipteraLimoniidae

(Westwood, 1836)

EF46BDA6-669B-5CC3-9870-F48283AA5FA1

Caloptera nepalensis Westwood, 1836: 681. Type locality: Nepal.
Pterocosmus velutinus Walker, 1848: 79. Type locality: Nepal.
Lechria nepalensis Brunetti, 1918: 317. Type locality: Nepal (Katmandu).
Eriocera nepalensis Westw. (= *velutina*, Walk.): Edwards 1921: 76.
Pterocosmus velutinus (Erioceranepalensis): Edwards 1921: 99.
Lechria nepalensis : Edwards 1924: 301.
Trichoneura (Xipholimnobia) nepalensis : Alexander 1963: 24; Alexander 1968: 95.
Hexatoma (Eriocera) nepalensis : Mitra et al. 2006: 234; Men and Yu 2015: 165.

##### Specimens examined

. 1 male (CAU), China: Xizang, Chayu, 1570 m, 1978.VI.25, Fasheng Li. 1 male (CAU), China: Xizang, Chayu, 1700 m, 1978.VI.26, Fasheng Li. 1 male (CAU), China: Xizang, Beibeng, 2014.VII.27, Yan Li. 1 female (CAU), China: Xizang, Chayu, 2014.VIII.3, Yan Li. 1 male, 2 females (CAU), China: Xizang, Linzhi, Gongbujiangda, Ganglangcun. ***Holotype***: male, Nepal, Hardwicke Bequest; accession no. NHMUK010397658, BMNH(E)247599 (BMNH).

##### Diagnosis

. Antenna has 8 segments. Wing is brown, but yellowed at basal 1/5; anal cells are much paler; a white discal area is before cord, including cell *R_1_* to cell *CuA_1_*; *m-cu* is near 2/3 of cell *dm*. Posterior margin of tergite 9 is produced; interbase is triangular, stubby at base.

##### Redescription

. Male (*N* = 4): Body length 12.5–22.5 mm, wing length 12.4–15.5 mm, antenna length 3.3–3.8 mm.

**Figure 9.**
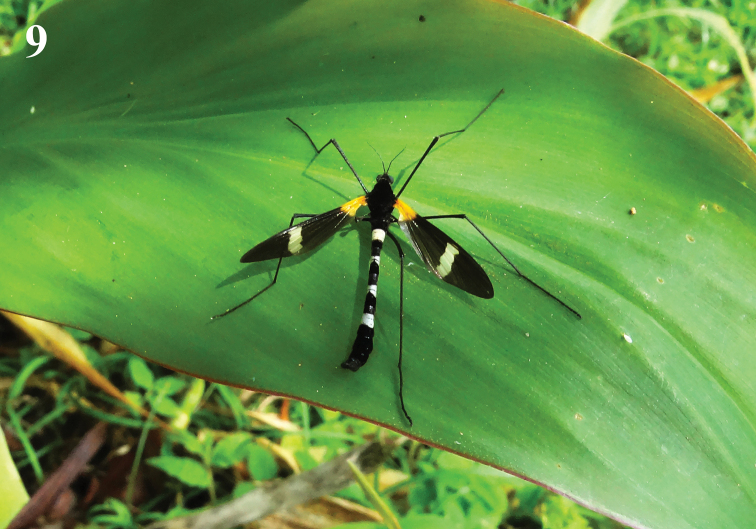
Hexatoma (Eriocera) nepalensis (Westwood, 1836), male. Photo by Qicheng Yang.

**Figures 10–16.**
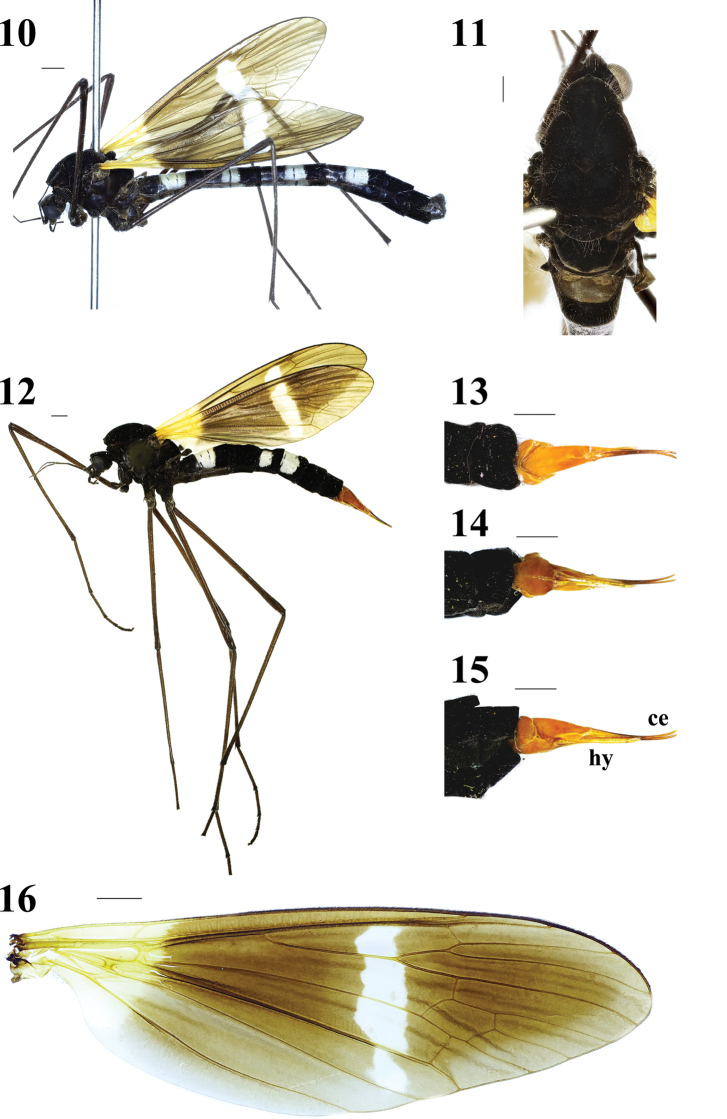
Hexatoma (Eriocera) nepalensis (Westwood, 1836) **10** male habitus, lateral view **11** male head and thorax, dorsal view **12** female habitus, lateral view **13** ovipositor, dorsal view **14** ovipositor, ventral view **15** ovipositor, lateral view **16** male right wing. Scale bars: 1 mm (10–16).

Head (Figs 9–11) velvet black with black setae. Rostrum and palpus blackish brown. Antenna 8 segmented, blackish brown with brown setae.

Thorax (Figs 9–11) velvet black with blackish brown setae. Legs with blackish brown setae; coxae and trochanters velvet black with long setae; femora, tibiae and tarsi blackish brown. Wing (Figs 9–10, 16) brown, basal 1/5 yellowed, anal cells much paler; a white discal area before cord, including cell *R_1_* to cell *CuA_1_*; veins brown, more yellowed in brightened areas. Venation: *R_2_* oblique, *R_2+3_* relatively short, shorter than *R_2_*; cell *M_1_* lacking; *m-cu* near 2/3 of cell *dm*. Halter (Figs 9–11) length approximately 2.1 mm, grayish brown with blackish brown setae.

Abdomen (Figs 9–10) with short black setae. Segments 2–5 elongate, shining black at base, pale white gray in middle, velvet black at tip; segments 1 and 6–7 velvet black; segment 8 shining black.

**Figures 17–21.**
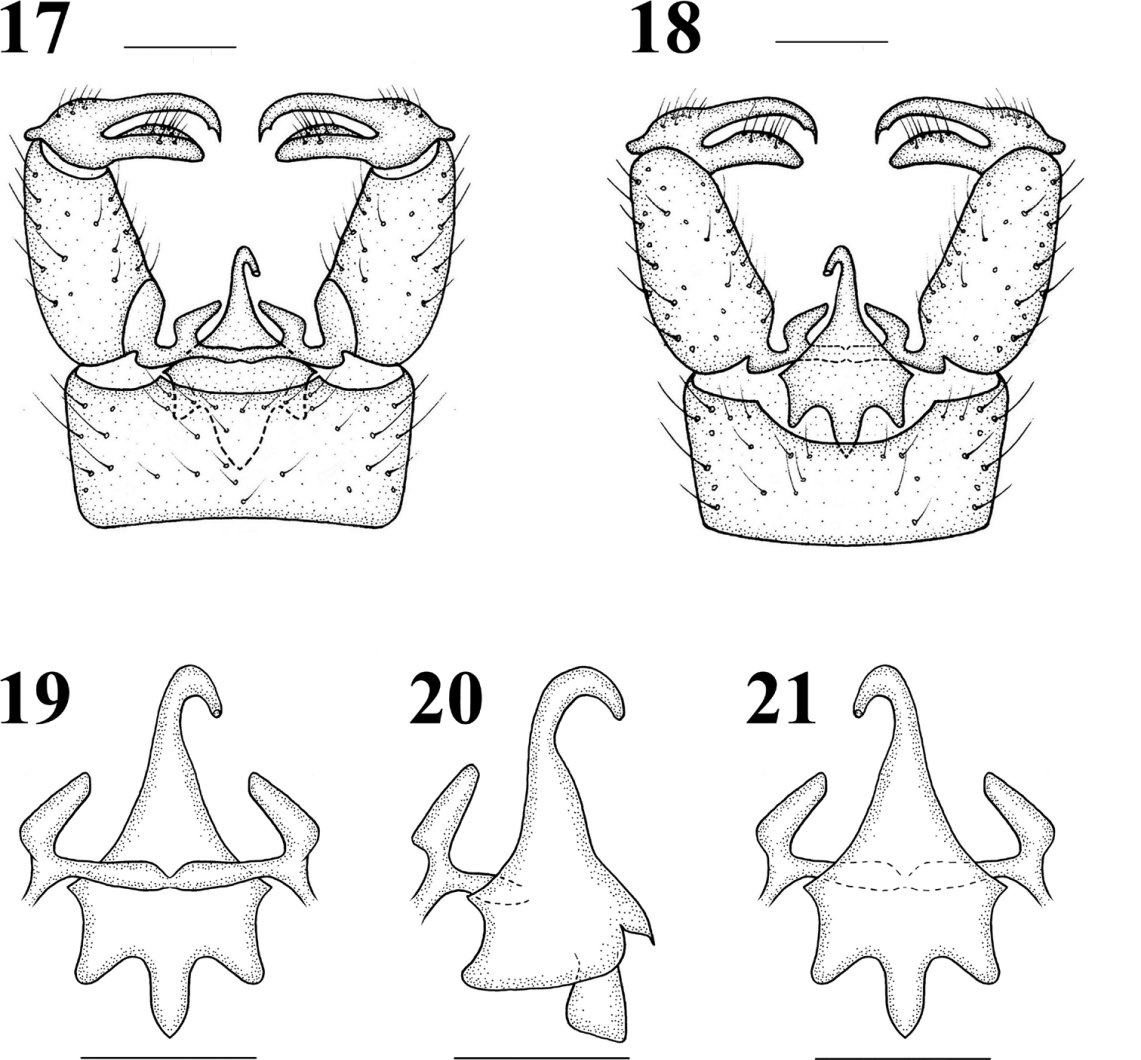
Hexatoma (Eriocera) nepalensis (Westwood, 1836), male **17** terminalia, dorsal view **18** terminalia, ventral view **19** aedeagal complex, dorsal view **20** aedeagal complex, lateral view **21** aedeagal complex, ventral view. Scale bars: 0.5 mm (17–21).

Male terminalia (Figs 9–10, 17–21) shining black with blackish brown setae. Posterior margin of ninth tergite produced, its margin concave; posterior margin of ninth sternite with a deep U-shaped shallow; gonocoxite moderately stubby; clasper of gonostylus with long setae at base, slender, terminal spine decurved; lobe of gonostylus short and stout, terminal margin swollen with long setae; interbase triangular, stubby at base; aedeagus longer, apically directed ventrally.

**Female** (*N*=3): Body length 17.3–19.4 mm, wing length 13.3–15.4 mm, antenna length 3.6–4.4 mm.

Female (Figs 12–15) resembles male. Abdomen shorter; tergites 2, 4–5 pale white gray at base, velvet black at tip; tergites 1, 3, 6–7 velvet black; sternites 1–7 velvet black; segment 8 velvet orange.

Ovipositor (Figs 12–15) elongate, velvet orange. Cercus narrowed toward tip. Hypogynial valve shorter, narrowed toward tip.

##### Distribution

. Afghanistan; China (Sichuan, Guangdong, Xizang), India (Assam and/or Arunachal Pradesh), Malaysia (Peninsula), Nepal.

##### Remarks

. This species is here recorded from Xizang, China for the first time.

#### Hexatoma (Eriocera) paragnava

Taxon classificationAnimaliaDipteraLimoniidae

Alexander, 1973

7178BB81-9F29-5EBF-A15E-668B11802478

Hexatoma (Eriocera) paragnava Alexander, 1973: 7. Type locality: India, Assam.

##### Specimens examined

. 19 males, 2 females (CAU), China: Xizang, Motuo, Beibeng, Jiangxincun, 800 m, 2019.V.29, Qicheng Yang (light trap). 1 female (CAU), China: Xizang, Motuo, Beibeng, Jiangxincun, 800 m, 2019.V.30, Qicheng Yang (light trap). 4 males, 3 females (CAU), China: Xizang, Motuo, 2019.V.31, Qicheng Yang (light trap).

##### Diagnosis

. Front and mouth parts are very reduced; antenna is very long and 6-segmented; vertical tubercle is very large. Wing is pale yellow; *m-cu* is near 1/6 of cell *dm*. Posterior margin of ninth tergite has a U-shaped notch; interbase is two-layered membranous structure with spine-like apex.

##### Redescription

. Male (*N*=23): Body length 8.6–11.0 mm, wing length 10.2–15.0 mm, antenna length 35.5–47.0 mm.

**Figures 22–28.**
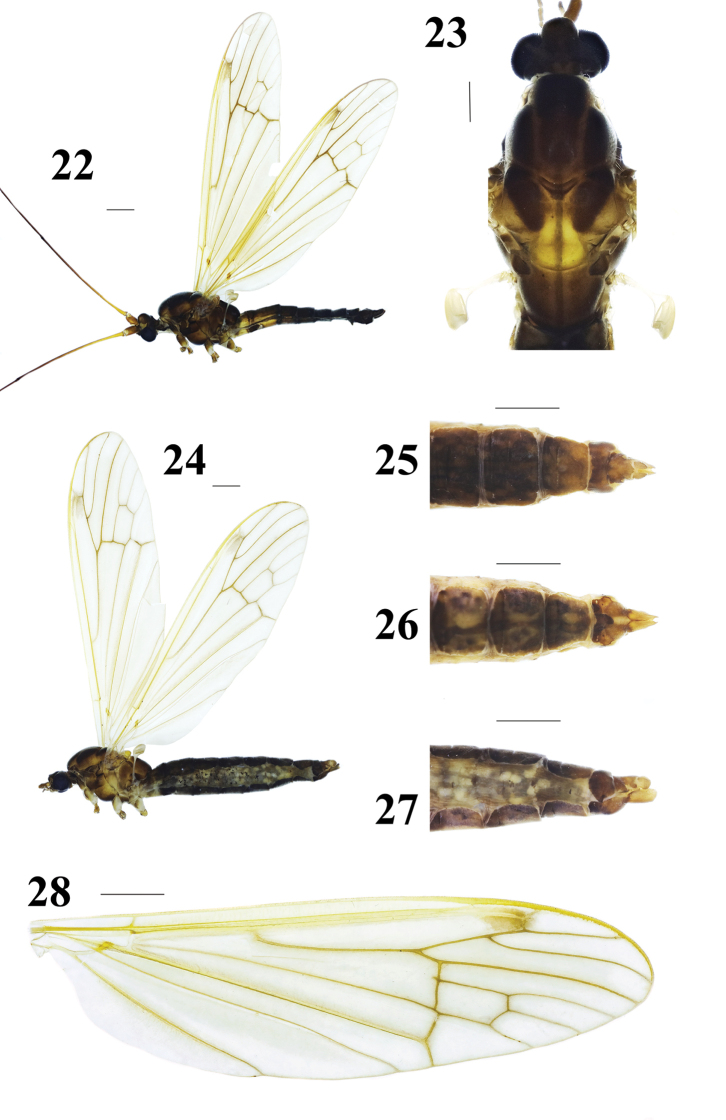
Hexatoma (Eriocera) paragnava Alexander, 1973. **22** male habitus, lateral view **23** male head and thorax, dorsal view **24** female habitus, lateral view **25** ovipositor, dorsal view **26** ovipositor, ventral view **27** ovipositor, lateral view **28** right wing. Scale bars: 1 mm (22–28).

Head (Figs 22–23) brown. Front and mouth parts very reduced, brownish yellow, palpus brown. Antenna 6-segmented, very long approximately three or four times as long as wing; scape and pedicel shorter, brownish yellow; flagellum very long, first flagellomere brownish yellow at base, brown at tip; remainder of flagellum brown with short blackish brown setae. Vertical tubercle more brown, very large, bulbous with setae on posterior aspect.

Thorax (Figs 22–23) brownish yellow to brown with gray setae. Pronotum brownish yellow; propleuron brown; prescutum dark brown with a broad brown stripe at middle; prescutal suture brown; scutum brownish yellow to brown; scutellum yellow with a slender brownish yellow stripe at middle; mediotergite brownish yellow to brown. Thoracic pleuron mostly brown throughout, except prescutum, anepimeron, katepisternum and metakatepisternum partly brownish yellow. Legs: coxae and trochanters brownish yellow with gray setae; femora and tibiae brownish yellow with short brown setae; tarsi brown with brown setae. Wing (Figs 22, 28) pale yellow; stigma and veins brownish yellow. Venation: *R_2_* moderately straight; *R_2+3_* near half of *R_2+3+4_*; cell *M_1_* lacking; *m-cu* near 1/6 of cell *dm*. Halter (Figs 22, 23) length approximately 1.9 mm, whitened gray.

Abdomen (Fig. 22) with brownish yellow setae. First two segments more yellowed at lateral margin; segments 3–8 dark brown.

**Figures 29–33.**
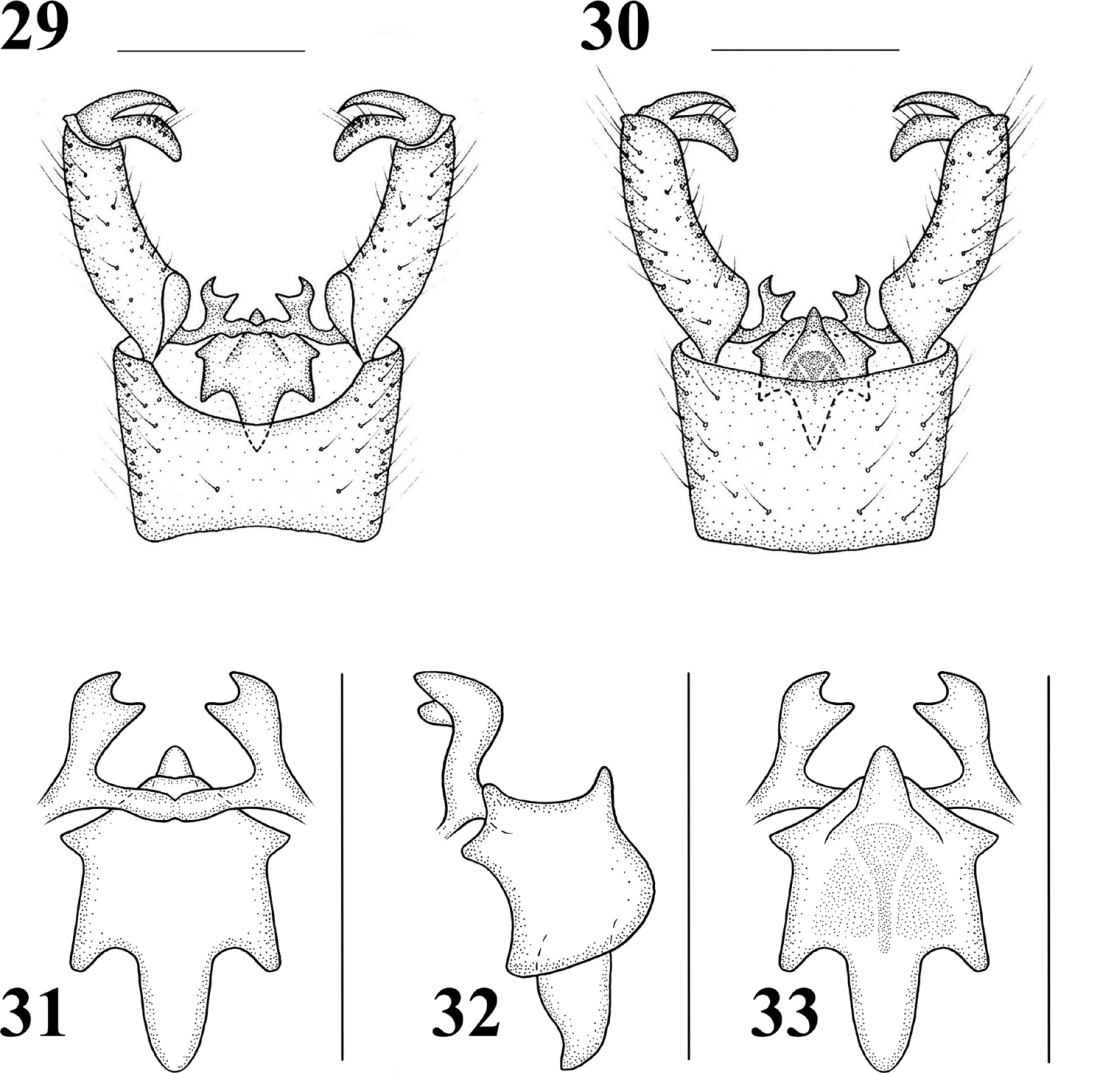
Hexatoma (Eriocera) paragnava Alexander, 1973, male **29** terminalia, dorsal view **30** terminalia, ventral view **31** aedeagal complex, dorsal view **32** aedeagal complex, lateral view **33** aedeagal complex, ventral view. Scale bars: 0.5 mm (29–33).

Male terminalia (Figs 22, 29–33) brown with brownish yellow setae. Posterior margin of ninth tergite with a U-shaped notch; gonocoxite large, elongate cylindrical, gently curved; clasper of gonostylus slender, terminal spine decurved; lobe of gonostylus short and stout, swollen with setae at middle; interbase two-layered membranous structure with spine-like apex; aedeagus smaller.

**Female** (*N*=6): Body length 9.3–13.2 mm, wing length 10.8–12.3 mm, antenna length 1.2–1.4 mm.

Female (Figs 24–27) resembles male. Thoracic pleuron more brownish yellow. Abdomen plump, dark brown.

Ovipositor (Figs 24–27) short and fleshy, brownish yellow; cercus oval; Hypogynial valve longer.

##### Distribution

. India (Assam); China (Xizang).

##### Remarks

. This species was known previously only from India. With the present contribution it is recorded from China for the first time.

#### Hexatoma (Eriocera) perhirsuta

Taxon classificationAnimaliaDipteraLimoniidae

Alexander, 1973

9AFF41CC-1698-5502-95D7-DE471C813413

Hexatoma (Eriocera) perhirsuta Alexander, 1973: 8. Type locality: India, Assam.

##### Specimens examined

. 1 male (CAU), China: Xizang, Sejilashan, 3200 m, 2013.VIII.2. 2 females (CAU), China: Xizang, Lulang, 2013.VII.28. 1 male (CAU), China: Xizang, Motuo, 80K, 2014.VII.23, Yan Li (light trap). 1 male (CAU), China: Xizang, Motuo, 80K, 2014.VII.31, Yan Li (light trap). 1 male (CAU), China: Xizang, Yigong, 2017.VIII.8, Qicheng Yang.

##### Diagnosis

. Rostrum is very short; antenna has 6 segments, very long; vertical tubercle is large bulbous. Wing is brownish yellow; *R_2+3_* is nearly three times length of *R_2+3+4_*; cell *M_1_* is present; *m-cu* is near 1/6 of cell *dm*. Posterior margin of ninth tergite has a deep U-shaped notch; interbase is well-developed, a two-layered membranous structure with spine apex.

##### Redescription

. Male (*N* = 4): Body length 16.0–18.3 mm, wing length 18.2–21.3 mm, antenna length 46.0–61.2 mm.

**Figures 34–40.**
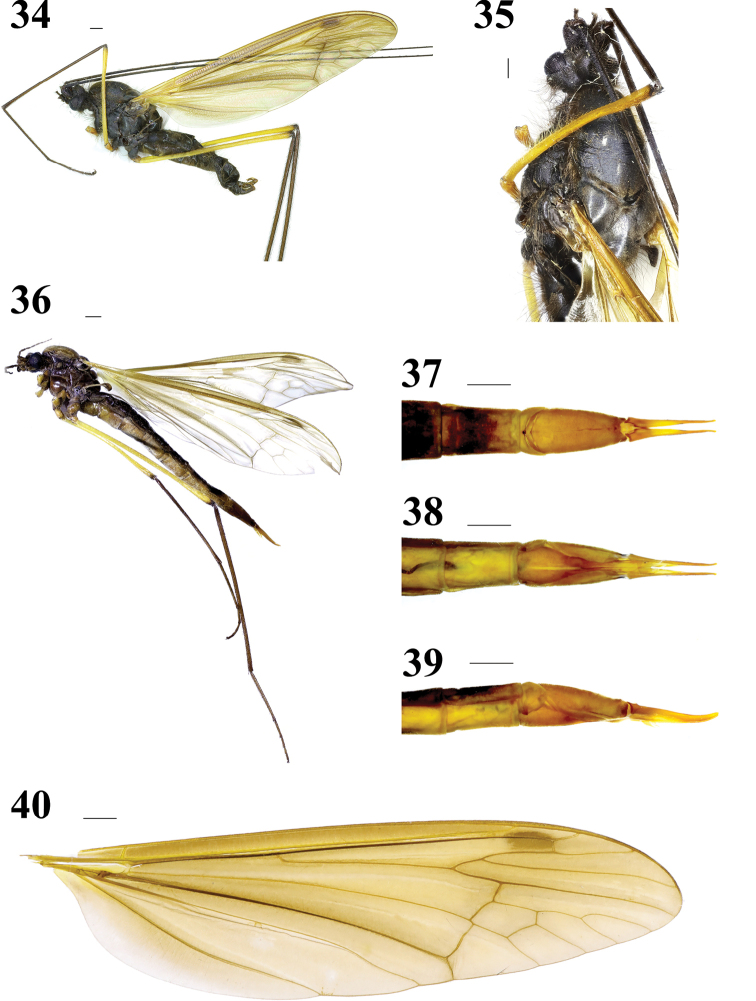
Hexatoma (Eriocera) perhirsuta Alexander, 1973 **34** male habitus, lateral view **35** male head and thorax, dorsal view **36** female habitus, lateral view **37** ovipositor, dorsal view **38** ovipositor, ventral view **39** ovipositor, lateral view **40** right wing. Scale bars: 1 mm (34–40).

Head (Figs 34–35) dark brown with very long abundant brown setae. Rostrum very short, dark brown; palpi brown. Antenna 6-segmented, very long, more than three times as long as wing; scape and pedicel blackish brown with long brown setae; flagellum dark brown with short brown setae. Vertical tubercle large bulbous, dark brown with abundant very long brown setae.

Thorax (Figs 34–35) dark brown with abundant very long brownish yellow setae. Legs: coxae dark brown with abundant very long brownish yellow setae; trochanters brownish yellow with long brownish yellow setae; femora yellow with short brownish yellow setae; tibiae and tarsi brown with short brown setae. Wing (Figs 34, 40) brownish yellow, stigma slightly brown; veins slightly brown, very inconspicuous against the ground. Venation: *R_2_* moderately straight; *R_2+3_* nearly three times as long as *R_2+3+4_*; cell *M_1_* slightly longer than its petiole; *m-cu* near 1/6 of cell *dm*. Halter (Figs 34–35) length approximately 3.2 mm, halter stem grayish brown with brown setae; knob brown.

Abdomen (Fig. 34) with abundant very long brownish yellow setae. First three segments brown; segments 4–8 dark brown.

**Figures 41–45.**
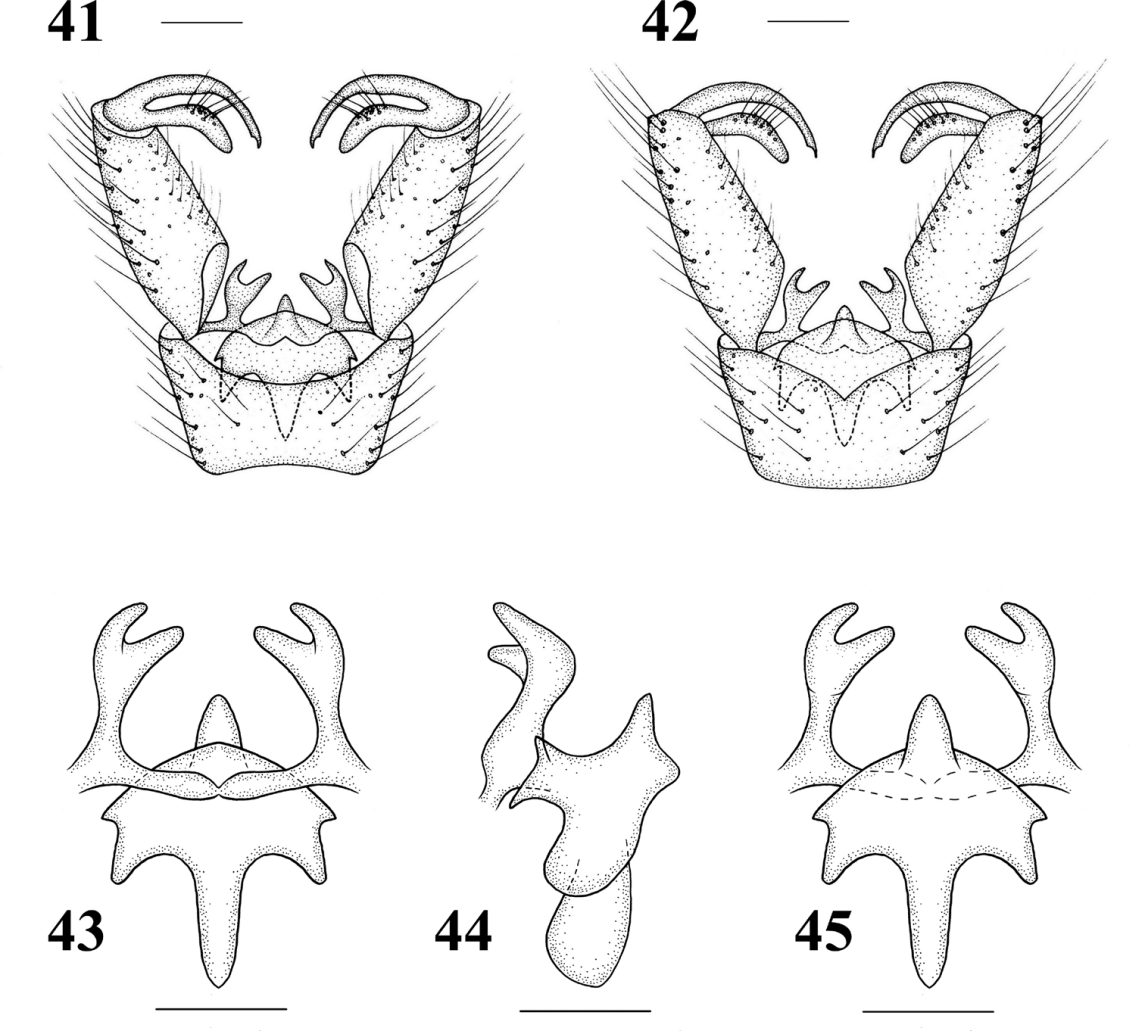
Hexatoma (Eriocera) perhirsuta Alexander, 1973, male **41** terminalia, dorsal view **42** terminalia, ventral view **43** aedeagal complex, dorsal view **44** aedeagal complex, lateral view **45** aedeagal complex, ventral view. Scale bars: 0.5 mm (41–45).

Male terminalia (Figs 34, 41–45) brown with long brownish yellow setae. Posterior margin of ninth tergite with a deep U-shaped notch; posterior margin of ninth sternite with a deep V-shaped shallow; gonocoxite large, elongate cylindrical; clasper of gonostylus long and slender, terminal spine decurved; lobe of gonostylus short and stout, swollen with setae at middle; interbase well-developed, a two-layered membranous structure with spine-like apex; aedeagus smaller.

**Female** (*N*=2): Body length 21.3–24.7 mm, wing length 19.6–22.5 mm, antenna length 3.1–3.5 mm.

Female (Figs 36–39) resembles male. Thorax more brownish yellow. Abdomen longer, venter more brownish yellow.

Ovipositor (Figs 36–39) elongate, reddish yellow. Cercus narrowed toward tip. Hypogynial valve shorter, narrowed toward tip.

##### Distribution

. India (Assam); China (Xizang).

##### Remarks

. This species was known previously only from India. This is the first record from China.

#### Hexatoma (Eriocera) xizangensis sp. nov.

Taxon classificationAnimaliaDipteraLimoniidae

C3912DE1-E135-5C01-9E41-CCE1EC224A3E

http://zoobank.org/72D7BB50-F2AB-40AC-AA95-C86E200960B1

##### Type material

**. *Holotype***: male (CAU), China: Xizang, Beibeng, Jiangxincun, 2019.V.30, Qicheng Yang (light trap).

##### Diagnosis

. Femora are yellow. Wing is brownish yellow with the following markings: an oblique transverse hyaline marking from *R* extended up to *CuA* in base of wing; origin of *Rs* with a small hyaline marking; a longer oblique transverse hyaline marking from *R* extended to wing margin before cord; both sides of *R_2_* with a hyaline marking; tip of *R_1_* from *Sc* to *R_3_* with an oblique transverse hyaline marking. *R_2_* is moderately oblique, approximately as long as *R_3+4_*, placed before fork of *R_3+4_*; cell *M_1_* is lacking; *m-cu* is near 2/3 of cell *dm*. Abdomen is brown to darker brown except segments 8–9 yellow. Posterior margin of ninth tergite has two small triangular processes; interbase is cylindrical, but stubby at base.

##### Description

. Male (*N* = 1): Body length 8.8 mm, wing length 7.2 mm.

**Figures 46–49. 46–48:**
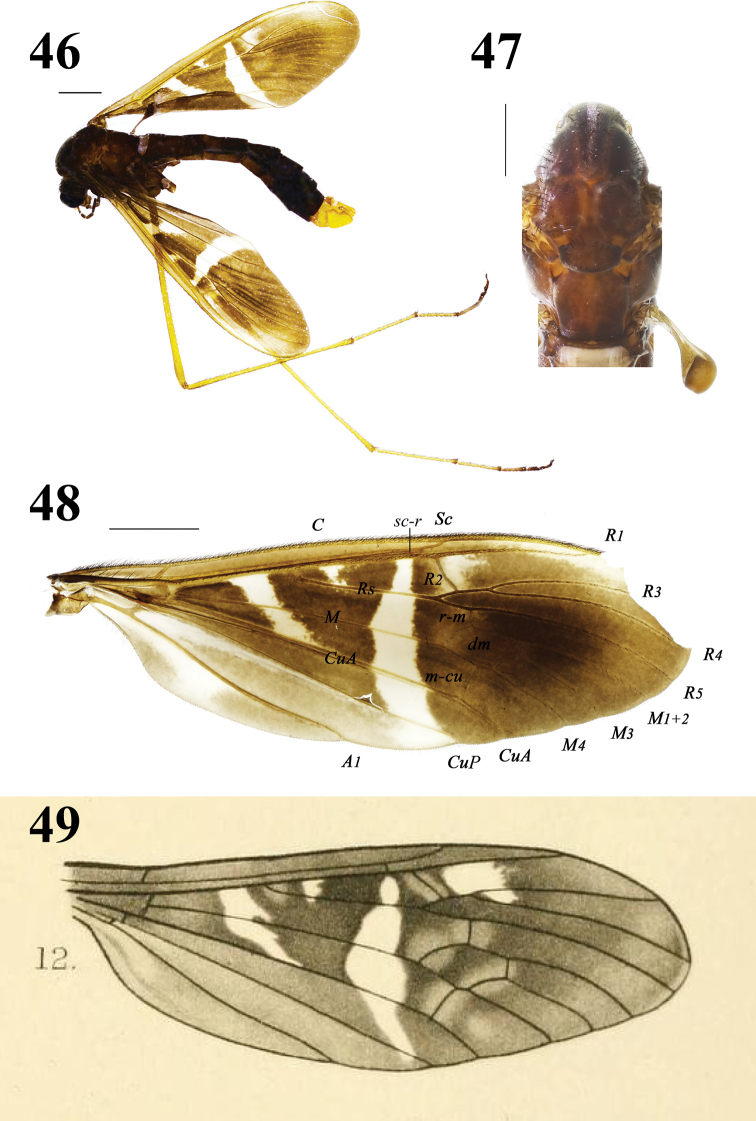
Hexatoma (Eriocera) xizangensis sp. nov., male **46** habitus, lateral view **47** male thorax, dorsal view **48** right wing **49** wing of Hexatoma (Eriocera) decorata (Brunetti, 1918: plate VII, fig. 12). Scale bars: 1.0 mm (46–48).

Head (Figs 46) brown with long brown setae. Rostrum very short, brownish yellow; palpi brown. Antennal scape brown with brown setae; pedicel brownish yellow; flagellum is broken.

Thorax (Figs 46, 47) brown with long brown setae. Legs: coxae and trochanters brown with long brown setae; femora and tibiae yellow with brown setae; basal two segments of tarsi yellow with brown setae, reminder segments brownish yellow to brown with brown setae. Wing (Figs 46, 48) brownish yellow, anal cells more yellow; anal cells with hyaline markings at base; an oblique transverse hyaline marking from *R* extended up to *CuA* in base of wing; origin of *Rs* with a small hyaline marking; a longer oblique transverse hyaline marking from *R* extended to wing margin before cord; both sides of *R_2_* with a hyaline marking; tip of *R_1_* from *Sc* to *R_3_* with an oblique transverse hyaline marking; veins slightly brown, very inconspicuous against ground. Venation: *R_2_* oblique, approximately as long as *R_3+4_*, placed before fork of *R_3_*_+_*_4_*; cell *M_1_* lacking; *m-cu* near 2/3 of cell *dm*. Halter (Figs 46, 47) length approximately 1.2 mm, pale brown.

Abdomen (Fig. 46) with brown setae. Segments 1–5 brown, segments 6–7 dark brown, segment 8 yellow.

**Figures 50–56. 50–54:**
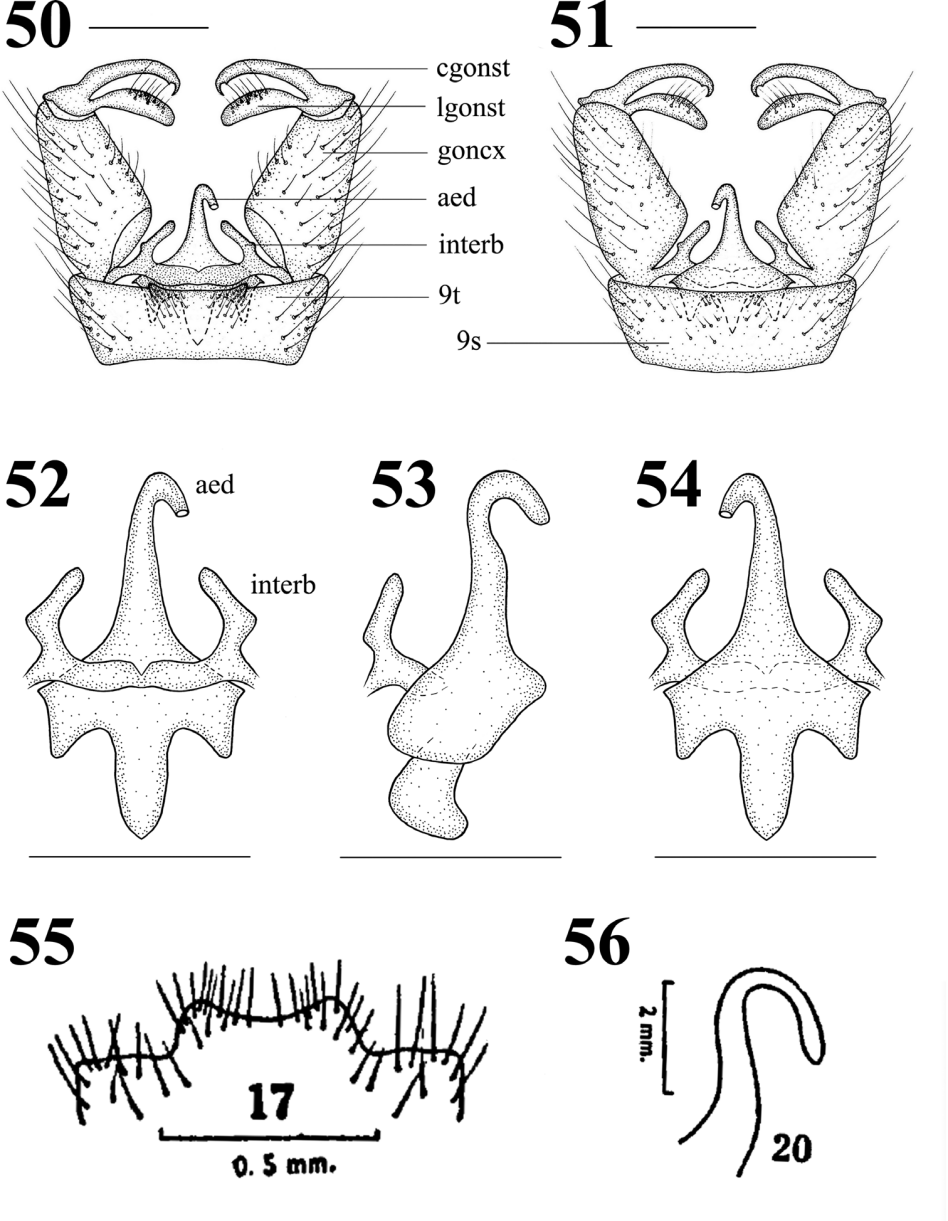
Hexatoma (Eriocera) xizangensis sp. nov., male **50** terminalia, dorsal view **51** terminalia, ventral view **52** aedeagal complex, dorsal view **53** aedeagal complex, lateral view **54** aedeagal complex, ventral view **55–56**Hexatoma (Eriocera) decorata (Brunetti, 1918), male. **55** ninth tergite (Joseph, 1977: 427, fig. 17) **56** aedeagus (Joseph, 1977: 427, fig. 20). Scale bars: 0.5 mm (50–54).

Male terminalia (Figs 46, 50–54) yellow with brownish yellow setae. Posterior margin of ninth tergite with two small triangular processes, with abundant brown setae; gonocoxite moderately stubby; clasper of gonostylus slender, terminal spine decurved; lobe of gonostylus short and stout, middle margin swollen with setae; interbase cylindrical, stubby at base; aedeagus longer, apically directed ventrally.

##### Female

. Unknown.

##### Distribution

. China (Xizang).

##### Etymology

. The species is named after Xizang Autonomous Region, where the type locality is located.

##### Remarks

. This new species is very similar to H. (E.) decorata (Brunetti, 1918) from India (W Bengal) in having similar wing markings, but can be separated from it by the wing with a longer oblique transverse hyaline marking from *R* extended to wing margin before cord (Figs 46, 48) and the posterior margin of the ninth tergite with two small triangular processes (Fig. 50). In H. (E.) decorata, the oblique transverse hyaline marking from *R* extends to the *CuP* before the cord (Fig. 49 i.e., Brunetti, 1918: plate VII. fig. 12; Edwards, 1924: 304; Joseph, 1977: 427, fig. 16) and the posterior margin of the ninth tergite is produced, its margin concave (Fig. 55 i.e., Joseph, 1977: 427, fig. 7). These two species are very special within the subgenus Eriocera, beCAUse of their unique position and slope of vein *R_2_* (Fig. 46, 48–49): it contacts vein *R_3+4_* (and not vein *R_3_* as is common in all *Eriocera* with a short vein *R_2+3+4_*), and is sloping forwards (also a very uncommon feature in *Eriocera*).

## Supplementary Material

XML Treatment for Hexatoma (Eriocera) latigrisea

XML Treatment for Hexatoma (Eriocera) nepalensis

XML Treatment for Hexatoma (Eriocera) paragnava

XML Treatment for Hexatoma (Eriocera) perhirsuta

XML Treatment for Hexatoma (Eriocera) xizangensis
